# Antimicrobial Efficacy of Intracanal Medicaments against *E. Faecalis* Bacteria in Infected Primary Molars by Using Real-Time PCR: A Randomized Clinical Trial

**DOI:** 10.1155/2020/6669607

**Published:** 2020-12-21

**Authors:** Yasamin Ghahramani, Najmeh Mohammadi, Ahmad Gholami, Dordaneh Ghaffaripour

**Affiliations:** ^1^Department of Endodontics, Shiraz Dental School, Shiraz University of Medical Sciences, Shiraz, Iran; ^2^Department of Pediatric Dentistry, Shiraz Dental School, Shiraz University of Medical Sciences, Shiraz, Iran; ^3^Pharmaceutical Sciences Research Center, Shiraz University of Medical Sciences, Shiraz, Iran; ^4^Department of Pharmaceutical Biotechnology, School of Pharmacy, Shiraz University of Medical Sciences, Shiraz, Iran; ^5^Department of Pediatric Dentistry, Shiraz Dental School, Shiraz University of Medical Sciences, Shiraz, Iran

## Abstract

**Aim:**

This study aimed to compare the antimicrobial efficacy of calcium hydroxide (CH) and triple antibiotic paste (TAP) against *E. faecalis* bacteria in infected primary molars.

**Methods and Materials:**

Thirty-nine 4–6-year-old children with an infected primary molar were randomly divided into three equal groups (*n* = 13) to receive either CH or TAP and an untreated control group. Following access cavity preparation, the first microbiological samples (S1) were collected by using absorbent paper points. The canals were prepared and rinsed. Then, CH or TAP was applied in the root canals. Seven days later, the second microbiological samples (S2) were collected. DNA extraction was performed to count *E*. *faecalis* bacteria by using real-time PCR for S1 and S2 samples. Data were analyzed through one-way ANOVA and Tukey's test (*α* = 0.05).

**Results:**

*E. faecalis* bacteria counts decreased significantly in CH and TAP groups compared with the control group (*P* ≤ 0.001). However, no statistically significant difference existed between these two groups (*P*=0.698).

**Conclusion:**

Both TAP and CH have significant antimicrobial effects as intracanal medicament between the treatment sessions in infected primary teeth.

## 1. Introduction

Bacteria are known as a major cause of pulpal and periapical diseases [1, 2]. The complex nature of the root canal system necessitates a combination of mechanical instrumentation and irrigation to reduce the microorganisms in the root canal [3]. It is quite difficult to entirely disinfect the root canal system in chronically infected primary molars. Chemomechanical preparation is often not sufficient, and many bacteria might remain [4, 5]. Recently, antimicrobial medicaments have been introduced to accomplish complete sterilization of root canals and yield better treatment outcomes [6, 7].

Calcium hydroxide (CH) is commonly used in endodontics as an intracanal medicament between the treatment sessions for necrotic permanent teeth [8, 9]. However, it does not affect some bacterial strains such as *E. faecalis* [9–11]. Infections in the primary root canals are associated with a broad variety of microorganisms, among which *E. faecalis* is one of the most prevalent and resistant bacteria species in both deciduous and permanent root canals [8, 12–14].

The need to wipe out deeply penetrated resistant bacteria from the dentinal tubules and improve the treatment of infected teeth have resulted in introduction of other medicaments such as triple antibiotic paste [7], which has been successfully used in primary and permanent necrotic canals [15, 16]. Application of triple antibiotic paste (TAP) as an intracanal medicament was observed to completely sterilize the infected canals within two weeks, proving to be certainly better than a single agent such as CH [7, 8, 17]. Higher success rate was reported in primary teeth treated with TAP compared with conventional pulpectomy [7]. Another study asserted TAP to be more effective than CH + normal saline as an intracanal medicament against *E. faecalis* [18]. Other medicaments including double antibiotic paste, TAP, and CH were found to prevent the growth of *E*. *faecalis* more effectively than rinsing with distilled water or NaOCl [19]. Given the limited relevant clinical data in primary dentition, the purpose of this study was to compare the antimicrobial efficacy of TAP and CH against *E*. *faecalis* bacteria in two appointment pulpectomy treatment of infected primary teeth by using the real-time PCR method.

## 2. Materials and Methods

### 2.1. Patient Selection

This clinical trial was approved by the local Ethics Committee of Shiraz University of Medical Sciences (IR.SUMS.REC.1397.828) and Iranian Registry of Clinical Trials (IRCT36341). Written informed consent was obtained after explaining the aim of the study to the patient's parents/guardians.

The subjects were 39 children (39 teeth), aged 4–6 years with infected maxillary or mandibular primary molars, considering a two-tailed significance level of 5% and 80% power. Exposure of the pulp with caries and fistula was determined in clinical diagnosis. The exclusion criteria were any radiographic sign of internal or external root resorption, excessive looseness of the tooth, involvement of permanent tooth bud, and the history of taking antibiotics within the preceding month.

The patients were randomly allocated into three groups (*n* = 13 per group) to receive either calcium hydroxide paste (1 gm CH mixed with 1 mL distilled water) or triple antibiotic paste (equal shares of ciprofloxacin, metronidazole, and minocycline mixed with normal saline) as the intracanal medicament. Based on the previous similar in vivo studies [7] and concerns about the cytotoxic effects of formocresol on the periapical area [20], the negative control group with no medicament (rinsing with normal saline and using a piece of sterile cotton pellet in the pulp chamber between the treatment sessions) was considered to compare the pure antimicrobial effect of intracanal medicaments. The group allocation was performed by an assistant not directly involved in the study after each patient was seated for the first appointment. The patients and the data evaluators were blinded to the type of the employed intracanal agent.

### 2.2. Collection of Microbiological Samples

The operation field was prepared, and scaling of hard and soft deposits was performed and cleansed with pumice; then, rubber dam was applied, and standard endodontic procedure was performed. Following access cavity preparation, the orifices of the larger canals (distal in mandibular molars and palatal in maxillary molars) were widened by using an orifice opener (Neolix, France) to facilitate easy entry of paper points deep into the canals. First microbiological samples (S1) were collected by inserting a sterile absorbent paper point #20 (Roeko GmbH and Co., Germany), leaving for about 30 seconds and then transferring it into a test tube containing 2 mL of brain heart infusion (BHI broth). The canals were prepared with K-file #20 and 25 (Mani, Japan) and rinsed with normal saline.

**Figure 1:**
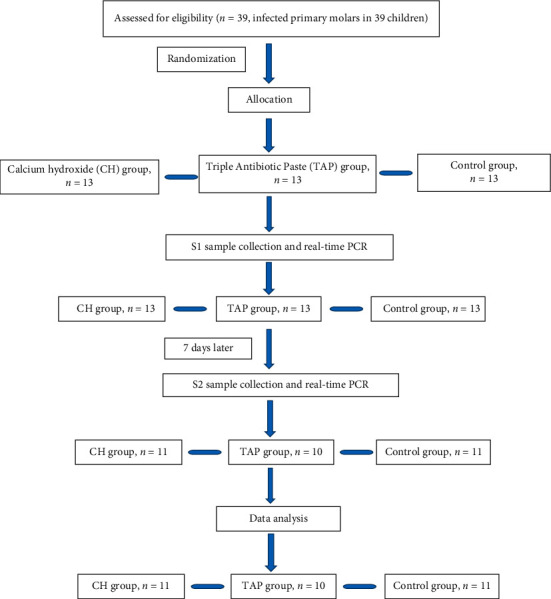
Diagram of the clinical procedure of the study.

Then, either CH or TAP was applied by using a size 25 lentulospiral (Henry Schein, Melville, USA), and the cavity was temporarily sealed with reinforced zinc oxide eugenol (Kemdent, England). After seven days, the temporary restoration was removed, and the canals were irrigated with normal saline to wash out the medicament, and second bacteriological samples (S2) were collected. The canals were then obturated with Metapex (Meta Dental Co. Ltd., Korea), and the teeth were restored with glass ionomer cement (GC Fuji IX, Japan) followed by cementation of a stainless steel crown (3M, USA).  Figure 1 displays the diagram of the clinical procedure.

### 2.3. Microbiological Procedures

The test tubes containing microbiological samples were preincubated at 37°C for 24 hours and shaken vigorously in a mixer (Vortex, Scientific Industries Inc., USA) for 60 seconds. Having confirmed similar weights of the tubes by using an electronic balance (AX200; Shimadzu Corp, Japan) and transferred to tryptic soy broth. The aliquots solutions were analyzed by quantitative real-time polymerase chain reaction (PCR, BioRad, Hercules, USA) to obtain the threshold cycle value of the samples. The microbial count was achieved via real-time PCR as follows.

### 2.4. DNA Extraction and Primer Design

The genomic DNA was isolated by using a bacterial genomic DNA isolation kit (CinnaPure DNA Gram + bacteria, Promega, USA). The concentration and purity of the DNA was determined through ultraviolet-visible spectrophotometry. The pure DNA extracted from *E. faecalis* was decimally diluted in a sterile Tris-EDTA buffer (pH = 8) and stored at −20°C until further use. Allele ID software was used to amplify a 138 base pair fragment of the gelatinase gene as a target sequence for the primer design. Gelatinase gene is a significantly predominant virulence factor gene in *E. faecalis* species and expresses better in the biofilm-positive strains [21–23]. The sequences of forward and reverse primers for the *E. faecalis* gelatinase gene were as follows: F (5' ACA CCA TTA TCC AGA ACT TAG GC 3') and R (5' GCT GCT GAC ACC ACT GAA G 3'). The specificity of the primer sequences was aligned and compared by using the BLAST algorithm in the GenBank database of NCBI.

### 2.5. Efficiency of the Reaction and Determination of the Linearity

Negative PCR controls (no DNA template) and negative qPCR amplification controls (*Enterococcus faecalis* total DNA template) were prepared in duplicate alongside the experimental total DNA sample to normalize any background signal obtained following amplification. To estimate the number of *E. faecalis* and total bacteria in samples, total DNA standards were prepared from an *E. faecalis* laboratory strain by using 10-fold serial dilutions ranging from 10 ng/_l to 100 fg/_l or DNA from *E. faecalis.* The strain was purified within a concentration range of 10^1^–10^6^ genomic DNA copies per reaction, and the linearity and efficiency of the PCR assay were determined. One microliter of each dilution was added to five replicas in PCR tubes and run the real-time PCR machine. The efficiency of the gelatinase gene-based real-time PCR assay was calculated by using the following formula: efficiency = [10^(−1/slope)^]^−1^.

### 2.6. Real-Time PCR Conditions

Real-time PCR and data analysis were performed in the iCycler iQ real-time detection system (BioRad laboratories) using RealQPCR 2^*∗*^Master Mix (SYBR Green), Ampliqon. PCR conditions were 95°C for 5 min and then 50 cycles consisting of 95°C for 15 s, 60°C for 30 s, and 72°C for 20 s. The melting curve (Tm) analysis of the final PCR product was carried out from 50°C to 90°C at 1°C intervals. An automatic threshold setting of 0.2 was used for all samples. A positive reaction and a nontemplate reaction were included in all tests. The standard curves were constructed from a dilution series with a concentration range (2 × 10^6^–2 CFU/ml or g) of bought sample. DNA was extracted, quantified, and amplified. Then, the standard curves were constructed. The correlation coefficient (*R*^2^) and efficiency of the amplification were calculated.

### 2.7. Statistical Analysis

All analyses were carried out by using SPSS software (version 20, SPSS Inc., USA). One-way analysis of variance (ANOVA), followed by Tukey's test and the *t*-test, was used to compare the results of real-time PCR between the groups at S1 and S2 (*α* = 0.05).

## 3. Results

*E. faecalis* was prevalent in almost 82% of the patients (*n* = 32 teeth), on which the statistical analyses were performed. Due to the high range of bacterial counts, log transformation of the counts was performed to normalize the data before statistical evaluation.  Table 1 shows the mean and standard deviations (SD) of *E. faecalis* counts in all groups at S1 and S2. One-way ANOVA showed that the mean counts of *E. faecalis* were homogeneous before instrumentation (S1) with no statistically significant difference (*P*=0.123). However, the three groups were significantly different at S2 (*P* ≤ 0.001).

According to the results of Tukey's post hoc test, *E*. *faecalis* bacterial counts decreased significantly in treatment groups compared with the control group (*P* ≤ 0.001). Yet, the two treatment groups (TAP and CH) were not significantly different (*P*=0.698). Comparing S1 and S2 through the *t*-test revealed the highest and lowest reduction of mean numerical *E*. *faecalis* counts to be related to the TAP and the control group, respectively (Table 2).

## 4. Discussion

Despite several years of clinical research, successful treatment of necrotic primary teeth remains challenging. To determine an antimicrobial biocompatible material as an intracanal medicament, various decontamination protocols have been suggested in pediatric dentistry [24–26]. Undoubtedly, the results of in vitro studies do not fully conform to the clinical situations, since the experimental models cannot reproduce the optimum contact between medication and dentin and the involving variables [27]. Hence, the present study was one of limited pioneer studies because of its clinical nature on deciduous teeth.

Clinical evaluations of the antimicrobial efficacy of intracanal medicaments are usually performed on *E*. *faecalis* bacteria, which is among the most resistant and prevalent microorganisms found in the root canal system with practical access to the genomic consequence and primer design [8, 12, 13]. Calcium hydroxide is widely used as an intracanal agent, known as the gold standard endodontic medicament that eliminates microbes that survive instrumentation. Its efficacy is related to the release of hydroxyl ions in an aqueous environment [27–29].

Faria et al. [30] conducted one of the first clinical studies on the use of an intracanal medicament in primary teeth. They evaluated the antibacterial effect of mechanical preparation associated with irrigating solution and a CH paste in root canals of human primary teeth with pulp necrosis and apical periodontitis. That study asserted the necessity of topical application of an intracanal medicament in primary teeth. However, a more recent study showed that none of the commonly used intracanal medicaments (such as Ca (OH)_2_, 1% CHX gel, and 1% metronidazole gel) could completely eliminate the aerobic and facultative anaerobic microorganisms from the root canal system of human primary teeth with necrotic pulp [27]. Ineffectiveness of these medicaments in complete elimination of microorganisms has opened new door of research in this regard.

*E. faecalis* bacteria were found to be resistant to calcium hydroxide, especially when the high pH was not maintained [18]. The root canal system is so complicated that seemingly cannot be completely disinfected with any single antimicrobial agent. Combination of antibiotics not only solves this problem but also seems to reduce the development of resistant bacterial strains [25, 26].

Studies also evaluated the antimicrobial efficacy of TAP (a combination of metronidazole, minocycline, and ciprofloxacin) against pathogens commonly found in the root canal system including *E*. *faecalis* [26, 29]. Dutta et al. [8] evaluated the efficacy of combination of medicaments (TAP, CH, and chlorhexidine) in comparison with CH per se against *E. faecalis* in necrotic primary teeth in clinical conditions, by culturing and counting colony-forming units. They found that a combination of antimicrobial agents used as intracanal medicament was definitely better than a single agent such as Ca (OH)_2_. Reddy et al. [7] also reported that the primary teeth treated with TAP were clinically more successful than the conventional pulpectomy.

Although some clinical studies documented the efficacy of intracanal medicaments in infected permanent teeth [5, 17, 31], efficient pulpectomy of bizarre and tortuous root canals is still a challenge in primary teeth. Moreover, the physiologic root resorption makes the endodontic treatment to be different in primary and permanent teeth. Hence, the findings on permanent teeth cannot be directly attributed to the primary teeth [20].

The present study is among the few in vivo studies [32] on the efficacy of intracanal medicaments (especially TAP) in infected primary teeth by using the PCR method. Based on our results, both TAP and CH significantly reduced the bacterial counts compared with the control group. Similar findings were reported by some other clinical and laboratory studies [25, 29, 33]. The current study also detected a significant reduction of bacterial count between S1 and S2 in all the study groups. Such a significant reduction in the control group (*P*=0.033) could be attributed to the clinical factors such as the immune system performance of the human body, function of rinsing with normal saline, and sensitivity of real-time PCR technique.

In a similar study by using the PCR method, Nagata et al. [34] concluded that both CH and TAP significantly reduced bacteria in immature permanent teeth; however, they observed no significant difference between the medicaments. Although the difference of intracanal medicaments was not statistically significant in the present study, CH displayed numerically higher bacterial counts at S2. A similar study attributed this lower performance to the increasing pH by CH, which triggers the genetic cascades, and consequently modifies the characteristic of bacterial cells. Bacterial adaptation makes them more resistant to alkaline challenge [35].

Several investigations reported the higher antimicrobial efficacy of TAP. Adl et al. [28] found that TAP significantly reduced the colony-forming units of *E*. *faecalis*; while CH had only a moderate antibacterial effect. Madhubala et al. [29] detected that TAP and propolis were more effective than CH against *E. faecalis*. The two aforementioned studies used agar well diffusion and MIC methods; while, the real-time PCR used in the present study is one of the most sensitive techniques for quantification of microbial species that are difficult to culture. The difference can also be due to the fact that the present study was an in vivo study on infected primary teeth.

Among the limitations of this study might be the use of paper point technique for taking samples, which was restricted to the main canals. Due to the morphology of primary root canals, sampling of all root canals with paper point before instrumentation would be impractical. Moreover, other bacterial causes of root canal infections in deciduous teeth were not evaluated since DNA extraction and primer design was possible just for *E. faecalis* bacteria. More in vitro and in vivo trials with larger sample sizes are needed on primary dentition to evaluate these antimicrobial effects on *E*. *faecalis* and other prevalent bacterial species found in the infected primary teeth root canal system. Further comparative studies are also suggested with a combination of TAP and CH.

## 5. Conclusion

Based on the present results, it was concluded that both TAP and CH have significant antimicrobial effects as intracanal medicament between the treatment sessions in infected primary teeth.

## Figures and Tables

**Table 1:** Mean and standard deviation of *E. faecalis* bacteria counts at S1 and S2.

Sampling stage	Groups	*N*	Mean	SD	*P* value
S1	CH	11	8.556	0.079	0.123
	TAP	10	7.141	1.517	
	Control	11	8.570	0.254	

S2	CH^*a*^	11	2.798	0.222	≤0.001
	TAP^*a*^	10	0.755	0.123	
	Control^*b*^	11	7.864	0.070	

^*∗*^Groups identified by different superscript letters were statistically significantly different, and groups with common superscript letters were not statistically significantly different.

**Table 2:** Comparison of the log transformation of the bacterial counts between S1 and S2.

Group		Mean	SD	*N*	*P* value
CH	S1	8.556	0.079	11	0.003
	S2	2.798	0.222	11	

TAP	S1	7.141	1.517	10	0.005
	S2	0.755	0.123	10	

Control	S1	8.570	0.254	11	0.033
	S2	7.864	0.070	11	

## Data Availability

The data used to support the findings of this study are available from the corresponding author upon request.
